# Geometry-aware point cloud clustering for spherical-component aggregate modeling

**DOI:** 10.1038/s41598-025-32832-y

**Published:** 2025-12-20

**Authors:** Yuta Muramatsu, Syuhei Sato, Kaisei Sakurai

**Affiliations:** 1 Faculty of Computer and Information Sciences, Hosei University, Tokyo, 184-8584 Japan; 2 Prometech CG research, Tokyo, 113-0033 Japan; 3 CyberAgent, Inc., Tokyo, 150-0042 Japan

**Keywords:** Modeling, 3D reconstruction, Point cloud, Aggregate, Engineering, Mathematics and computing

## Abstract

This paper proposes a method for obtaining independent mesh models of individual components from a point cloud representing an aggregate. An aggregate consists of a collection of small, similar components, such as individual grapes in a bunch. Typical shape reconstruction creates a rough shape of the entire bunch, but fails to recover individual components from the bunch due to occlusion and missing points for shapes. To achieve this type of modeling, we assume that each component can be approximated as a spherical shape. Leveraging this assumption, we develop geometry-aware clustering that identifies and segments individual components from the aggregate. During this procedure, we search for the optimal position and size of a predefined aggregate component that best fits the cluster. When overlapping components are detected, the corresponding clusters are merged. We demonstrate the effectiveness of the proposed method by applying it to several types of aggregates, such as grapes and tomatoes.

## Introduction

**Fig. 1:**
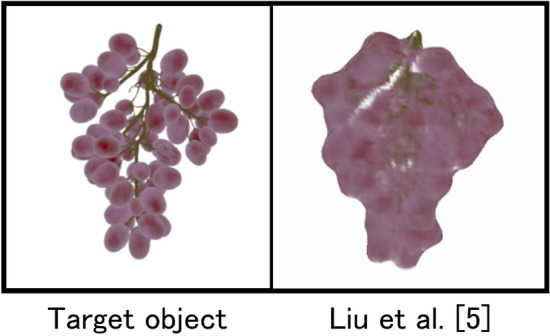
Reconstructed result using a previous approach (Liu et al.).

In computer graphics (CG), creating a variety of 3D objects is essential to enhance the realism of visual content. Since scenes typically contain a large number of objects, modeling all of them manually requires significant time and effort. To address this issue, various methods have been proposed for simplifying the process of 3D object modeling. In particular, many recent approaches allow users to easily model 3D objects by using images or illustrations as input ^1–6^. However, these methods typically generate a single mesh model per object. Therefore, it is difficult to model each individual component of an aggregate, such as the individual grape berries in a bunch, as an independent mesh. In our preliminary experiments, we applied the method proposed by Liu et al.^5^. to reconstruct a 3D model of a grape bunch. However, as shown in Fig. 1, the individual grape berries were not separated, and the result was a single fused shape lacking clear separation between components. More recently, methods based on Gaussian Splatting have attracted significant attention, and Cen et al. proposed a 3D segmentation method utilizing Gaussian Splatting^7^. However, even with these state-of-the-art techniques, it remains challenging to individually recognize each grape berry in a bunch.

To address this problem, we propose a method that models aggregates by reconstructing each component as an independent mesh. Specifically, we segment the point cloud obtained from images into individual components through a combination of clustering and component fitting. Our method takes as input a point cloud representing the target aggregate and a mesh model of a single component. The point cloud is generated from input images using COLMAP^1^, a widely used Structure-from-Motion pipeline. We apply k-means clustering to the point cloud, and for each cluster, we determine the position, orientation, and size of the best-fitting aggregate component via a search based on Chamfer distance. However, this process alone is not sufficient to accurately identify all components, as one component may be split into multiple clusters, or a single cluster may span multiple components. As a solution, we merge clusters based on the overlap between the fitted components, and then reapply the component fitting to the merged clusters. In this paper, we focus on aggregates composed mainly of spherical components, and demonstrate the effectiveness of our method on various inputs, including both synthetic CG images and real-world photographs.

As a line of research on 3D reconstruction of aggregates, several methods have been proposed in the agricultural domain for estimating the number of grape berries and evaluating their quality^8,9^. These methods aim to support harvesting and quality control by estimating the number and size distribution of grape berries from one or more input images. However, they tend to be computationally expensive and often require specialized equipment. Moreover, to the best of our knowledge, no prior work has focused on estimating the 3D information of individual aggregate components for the purpose of CG modeling.

## Related work

### 3D reconstruction

Structure-from-Motion (SfM) and Multi-View Stereo (MVS) are widely used techniques for reconstructing the 3D shape of objects from multi-view images. COLMAP by Schönberger et al.^1^ enables high-precision 3D reconstruction from images by detecting corresponding feature points across multiple views and estimating camera motion and 3D point positions based on geometric relationships. Langguth et al. achieved high-accuracy 3D reconstruction by incorporating shading information in addition to stereo matching^10^. Cui et al. proposed a reconstruction method that combines the advantages of incremental and global SfM approaches^11^. Hepp et al. developed a method specialized for large-scale building reconstruction using a drone^2^. While these methods are effective for generating high-quality 3D models of individual objects, they struggle to produce separated models of individual components when the target is a densely packed aggregate.

Recently, 3D reconstruction techniques based on deep learning have made remarkable progress. Mildenhall et al. proposed NeRF^3^, a method for synthesizing novel views from a set of images with known camera poses. NeRF represents a scene as a continuous 5D function and optimizes it using a neural network. Based on NeRF, several studies have explored novel view synthesis from a single input image^12^. However, these methods cannot represent geometry in the form of surface meshes. In response to this limitation, Tang et al. proposed a method for extracting surface meshes from NeRF^4^. Their approach generates high-quality models by adaptively refining vertex positions and face density on a coarse mesh extracted from NeRF, based on rendering errors. An approach that incorporates deep learning into Multi-View Stereo (MVS) has also been proposed. MVSNet, introduced by Yao et al.^13^, is an end-to-end architecture that replaces the depth map estimation process in MVS with deep learning. This method takes multi-view images as input and constructs a 3D cost volume representing depth hypotheses for each pixel to estimate the depth map. This approach enables faster and more accurate mesh generation compared to traditional MVS methods.

Furthermore, methods have been proposed to generate meshes directly without intermediate representations such as voxels or point clouds. Liu et al. proposed MeshDiffusion^14^, which directly generates 3D meshes using diffusion models. This method achieves mesh generation capable of representing finer undulations and sharp edges compared to conventional methods by learning data converted from object meshes in 3D shape databases into lattice structures composed of tetrahedra. GET3D by Gao et al.^15^ is a method that directly generates high-quality 3D textured meshes with arbitrary topologies by combining differentiable mesh representations with neural networks that predict color information from 3D spatial coordinates. These methods can create detailed mesh data with fewer images compared to existing SfM, but since they treat the target as a single object, it is difficult to obtain mesh models where the components of an aggregate are separated.

### Aggregate modeling

Several methods have been proposed for modeling aggregates. Ma et al. introduced a method for generating aggregates by synthesizing repeated elements over a large output region specified by the user, based on a small number of input samples^16^. By efficiently encoding and optimizing properties such as shape, size, color, and spatial distribution of the elements, this method produces visually plausible aggregates. Roveri et al. proposed a sample-based method for synthesizing repetitive structures^17^. This approach learns a given example pattern and applies it to new regions or shapes, generating visually consistent structures. Sakurai et al. proposed a modeling technique for aggregates with non-periodic stacked structures^18^. Their method generates aggregates solely through parameter control, by optimizing random element placements to minimize overlaps, without relying on physical simulations or sample data. Hsu et al. presented a system to assist with the interactive modeling of aggregates^19^. In their method, an element field is first defined based on partial user input regarding the position, orientation, and scale of elements. Then, the remaining elements are automatically generated by optimizing their positions, orientations, and scales to produce a coherent aggregate. These approaches allow the procedural modeling of visually plausible aggregates. Similarly, we aim to simplify the modeling of aggregates and propose a method that takes point clouds obtained from real images as input, enabling the generation of aggregates with spatial arrangements that more closely resemble real-world instances.

Although not intended for CG applications, methods related to aggregates have been proposed to reconstruct the three-dimensional shape of grapes, estimate the number of grape berries, and perform quality management^8,9^. These methods support harvesting and quality management by obtaining the distribution of grape numbers and sizes from one or multiple input images. However, these methods have constraints such as high computational cost and the need for specialized equipment. In contrast, our approach leverages the rapidly advancing techniques in computer vision to enable modeling using point clouds obtained from real-world images, without the need for specialized equipment. Furthermore, by combining clustering and element search on the point cloud, our method achieves a lower computational cost compared to the aforementioned approaches.

**Fig. 2:**
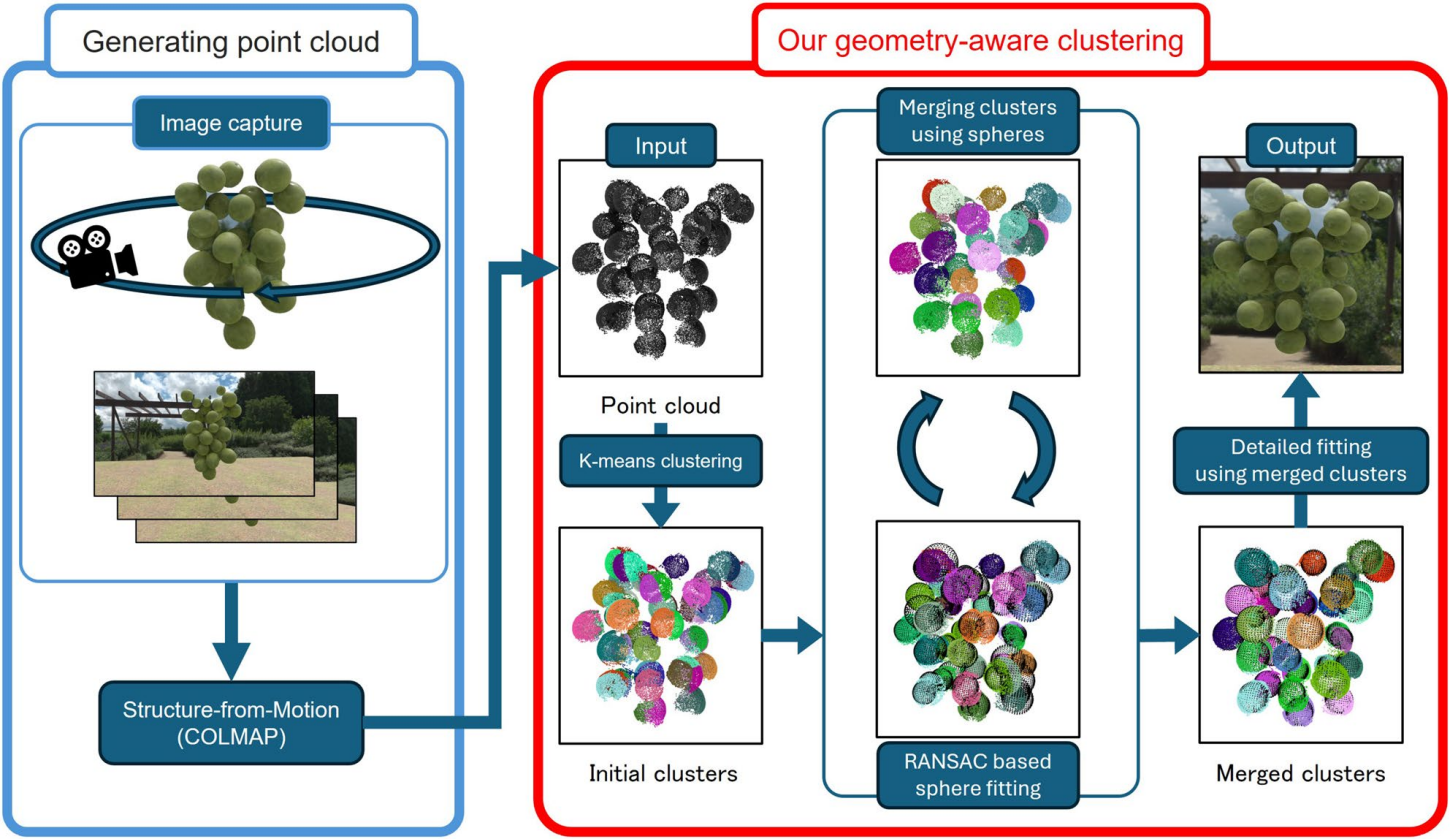
Overview of our framework.

## Our method

An overview of our geometry-aware clustering is shown in Fig. 2. In this study, the inputs are a point cloud representing an aggregate and a mesh model representing a single component (hereafter referred to as the component model). Since this work focuses on aggregates composed of spherical components, such as grapes and tomatoes, we use a sphere as the component model. The point cloud is generated using COLMAP^1^. Our method applies three processes to the input point cloud: clustering, component model fitting, and cluster merging, in order to segment the point cloud into clusters corresponding to individual components. Finally, we perform detailed fitting of the component model to each cluster, enabling component-wise modeling of the aggregate. The following sections describe each process in detail.

### Clustering point cloud

The point cloud representing an aggregate is obtained using an existing structure-from-motion method such as COLMAP. Since the reconstructed point clouds differ in scale depending on the target object and imaging conditions, each point cloud is normalized so that it fits within a bounding box of $[-1,1]$ along all axes. Next, to reduce computational cost, the normalized point cloud is downsampled using a voxel grid method. Specifically, the three-dimensional space is divided into a uniform grid of $200^3$ voxels, and all points contained within each voxel are replaced by a single point corresponding to their centroid. This reduction in point density effectively decreases the computational burden for the subsequent clustering and fitting processes, while preserving most of the geometric details of the aggregate. The preprocessed point cloud is then partitioned into clusters.

We adopt k-means clustering for this step. The k-means clustering is a method that partitions the given data into k clusters. Specifically, it begins by randomly initializing k cluster centroids (centers of mass), then assigns each data point to the cluster with the nearest centroid. The centroids are then recalculated based on the current cluster assignments, and the data points are reassigned to the nearest new centroids. This process is repeated until the centroids converge. The number of clusters is specified by the user as an approximate value that is larger than the expected number of components in the aggregate. This overestimation helps prevent a single cluster from spanning multiple components.

**Fig. 3:**
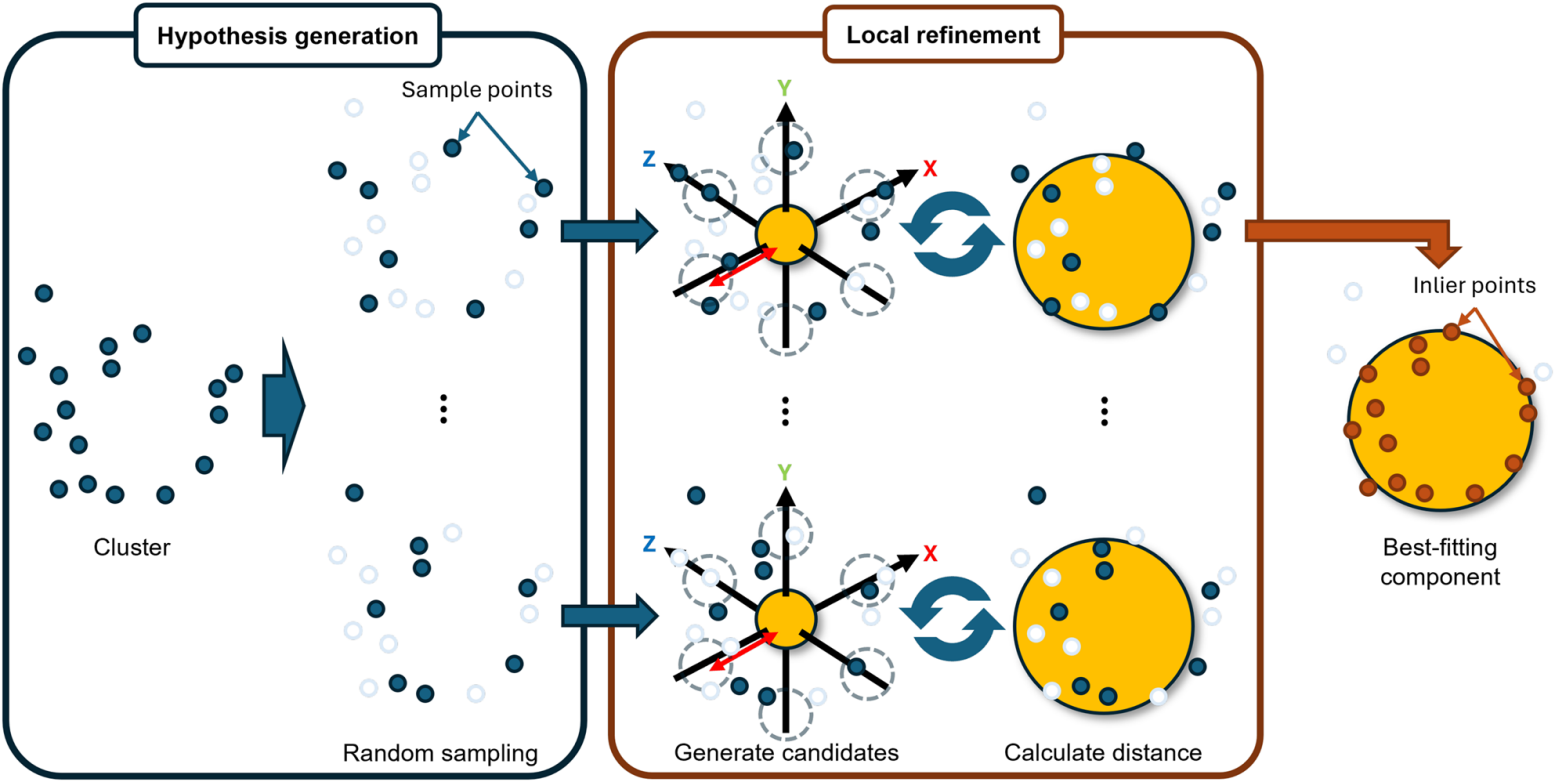
Overview of our fitting process.

### Fitting aggregate component to cluster

We fit a component model into each cluster in the clustered point cloud. An overview of this process is shown in Fig. 3. For each cluster, we perform a RANSAC-based search which consists of two steps: hypothesis generation and local refinement. In the hypothesis generation step, we randomly sample a small number of points from the cluster and compute a candidate sphere based on them. In the local refinement step, each candidate sphere is optimized using only the sampled points, refining its parameters (center and radius) to better fit those points. This process is repeated for multiple hypotheses, and the best-fitting sphere is selected based on Chamfer distance evaluated over the entire cluster. In our experiments, the number of hypotheses $N_{hypo}$ was set to 50, and the maximum number of iterations in the local refinement step $N_{refi}$ was set to 50. These values were determined empirically based on preliminary experiments.

In the hypothesis generation step, 10% of the points within each cluster are randomly sampled. For the sampled points, local refinement (described later) is performed to estimate the radius and center position of the sphere that best fits the sample. Then, for all points *p* in the cluster, the distance $d_p$ to the surface of the refined sphere is computed, and those points whose distances fall below a predefined threshold $\epsilon$ are marked as inliers. Specifically, this distance $d_p$ is computed as follows:

1 $$\begin{aligned} d_p = \left| r - \Vert \mathbf{x}_p - \mathbf{c} \Vert _2 \right| \end{aligned}$$

where, ${\mathbf{x}}_{{p}}$ is a position of the point *p*, $\mathbf{c}$ and *r* are the center and radius of a hypothesis sphere, respectively. We set $\epsilon =0.015$ for all examples. If the ratio of inlier points to the total number of points in the cluster exceeds 50%, the hypothesis is considered valid, and the Chamfer distance between the refined sphere and the inlier points is computed. This process is repeated for all hypotheses, and the hypothesis with the smallest Chamfer distance among the valid ones is selected as the best-fitting sphere for the cluster. Also, these parameters in the hypothesis generation step were determined empirically. Halving the parameters caused fitting failures, while doubling them increased runtime without improving results.

**Fig. 4:**
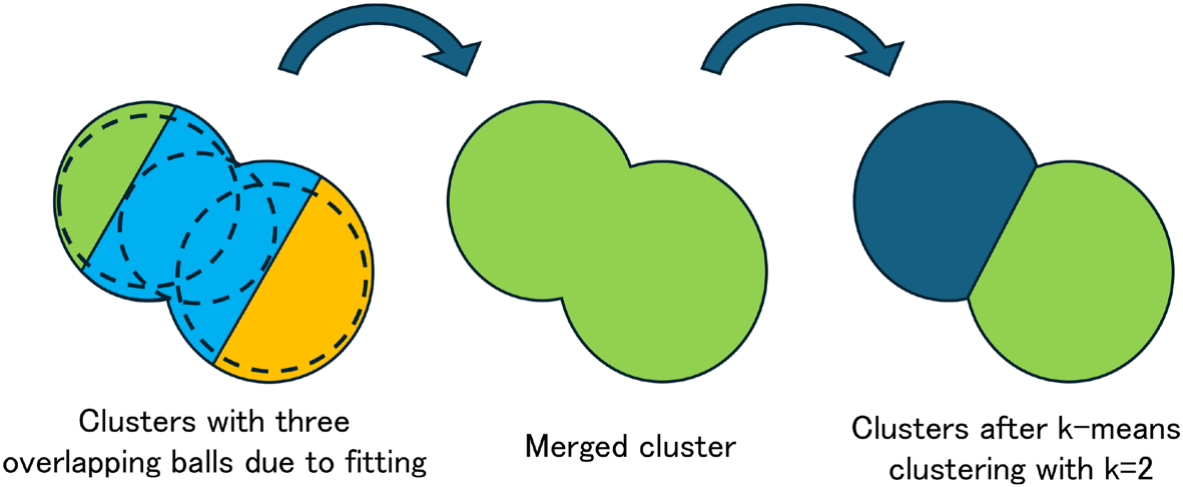
Cluster merging process.

In the local refinement step, the goal is to optimize the radius and center position of the sphere that best fits the sampled points within each hypothesis. First, a sphere with an initial radius specified by the user is placed at the centroid of the sampled points. Through experiments, we have confirmed that setting the initial radius to approximately match the smallest component in the target aggregate yields favorable results. Next, the method performs an iterative search by translating and scaling the sphere. Specifically, a set of candidate spheres is generated by perturbing the current sphere’s position and radius. For each candidate, the Chamfer distance to the sampled points is computed, and the candidate with the smallest distance is selected as the new reference sphere for the next iteration. We adopt a symmetric Chamfer distance $d_{CD}$, which serves as the objective function for this optimization:

2 $$\begin{aligned} d_{CD}(P_{smp}, V_{sph}) = \frac{1}{|P_{smp}|} \sum _{q \in P_{smp}} \min _{v \in V_{sph}} \Vert \mathbf{x}_q - \mathbf{x}_v \Vert _2 + \frac{1}{|V_{sph}|} \sum _{v \in V_{sph}} \min _{q \in P_{smp}} \Vert \mathbf{x}_v - \mathbf{x}_q \Vert _2 \end{aligned}$$

where, $P_{smp}$ is the set of sampled points and $V_{sph}$ is the set of vertices of the candidate sphere mesh. $\mathbf{x}_q$ and $\mathbf{x}_v$ indicate positions for the sampled points and the vertices of the candidate sphere, respectively. This process is repeated until one of the following conditions is met: (1) the Chamfer distance falls below a predefined threshold $d_{th1}$ (0.015 in our experiments), (2) the change in Chamfer distance from the previous iteration falls below a threshold $d_{th2}$ (0.001 in our experiments), or (3) the number of iterations reaches the maximum limit $N_{refi}$. The radius and position of the final sphere are taken as the optimized result. Each iteration evaluates 35 candidate spheres generated by combining 7 translations (including no translation) along the positive and negative directions of each of the x, y, and z axes (with a translation width of 0.01), and 5 radius scalings (0.8, 0.9, 1.0, 1.1, 1.2).

### Merging superfluous clusters

The k-means clustering does not always produce clusters that precisely follow the shape of individual components. As a result, it is possible for a single component to be split across multiple clusters, or for a single cluster to span multiple components. In such cases, the fitted spheres obtained in the earlier step often overlap with each other. To address this, we detect overlapping spheres and merge the corresponding clusters in order to improve the accuracy of component detection. In this study, two spheres are considered to be overlapping if the distance between their centers is less than 90% of the sum of their radii: $\Vert \mathbf{c}_i - \mathbf{c}_j \Vert _2 < \alpha (r_i + r_j)$, where $\mathbf{c}_i, \mathbf{c}_j$ and $r_i, r_j$ are centers and radii for i- and j-th spheres, and we set $\alpha = 0.9$. When such a pair is found, the corresponding clusters are merged. As shown in Fig. 4, if three or more spheres mutually overlap, all of their corresponding clusters are merged, and k-means clustering is reapplied using a new number of clusters equal to the number of overlapping spheres minus one. The fitting process described earlier is then repeated for the merged cluster. This process of overlap detection and cluster merging is repeated until no further overlapping spheres are found.

After the merging process, a more detailed fitting procedure is applied to each cluster using the position and radius of the best-fitting sphere obtained by the earlier RANSAC search as the initial values. Specifically, the sphere search process described in the previous local refinement step is extended to include per-axis scaling and rotation as additional parameters. The scaling operation is applied independently along each axis, analogous to the radius scaling described earlier. For rotation, seven variations are evaluated: six corresponding to $\pm {10^\circ }$ rotations around each of the x, y, and z axes, and one with no rotation.

**Fig. 5:**
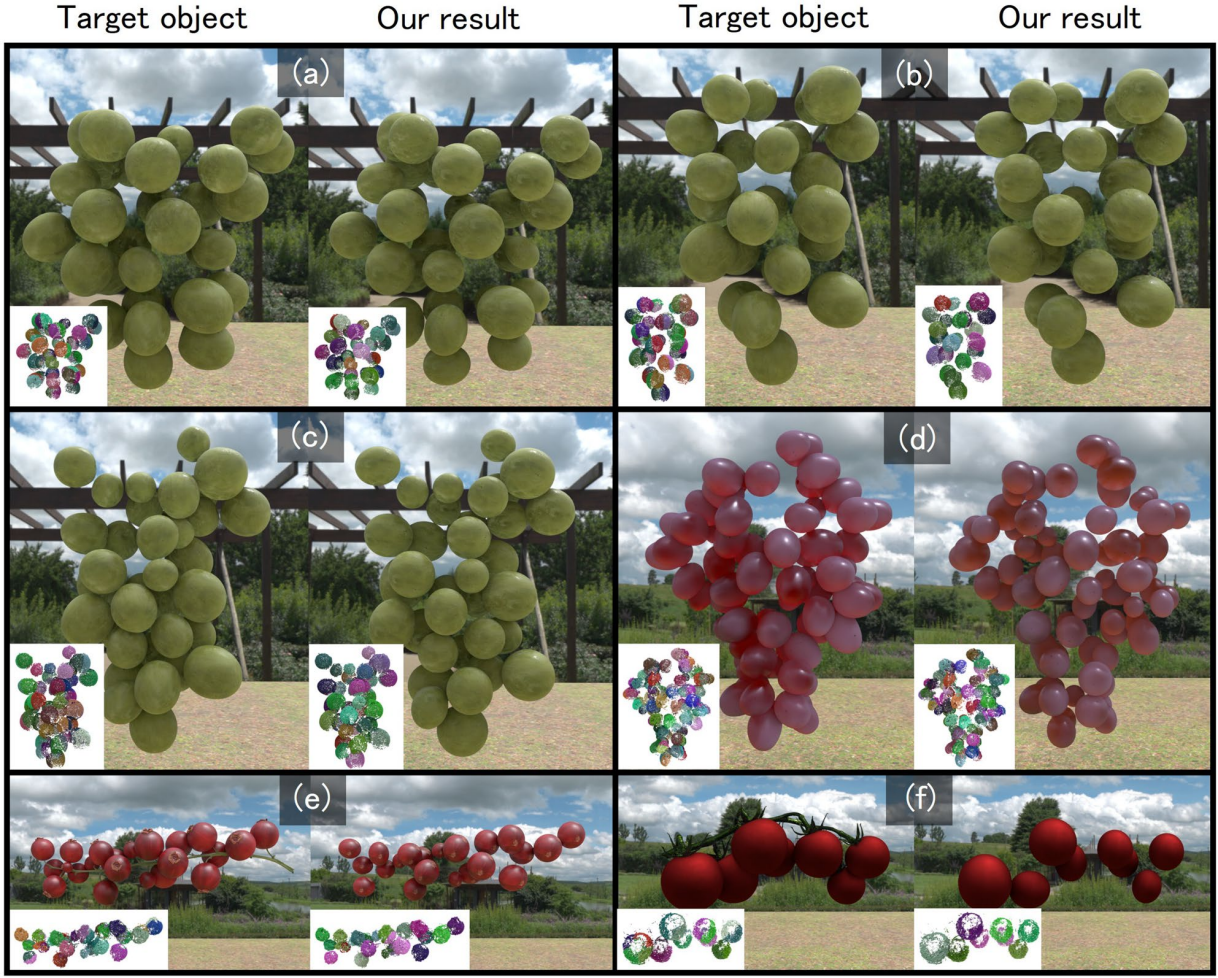
Our results on 3D models of aggregates. The inset image on the target object/our result shows initial/resultant clusters.

**Fig. 6:**
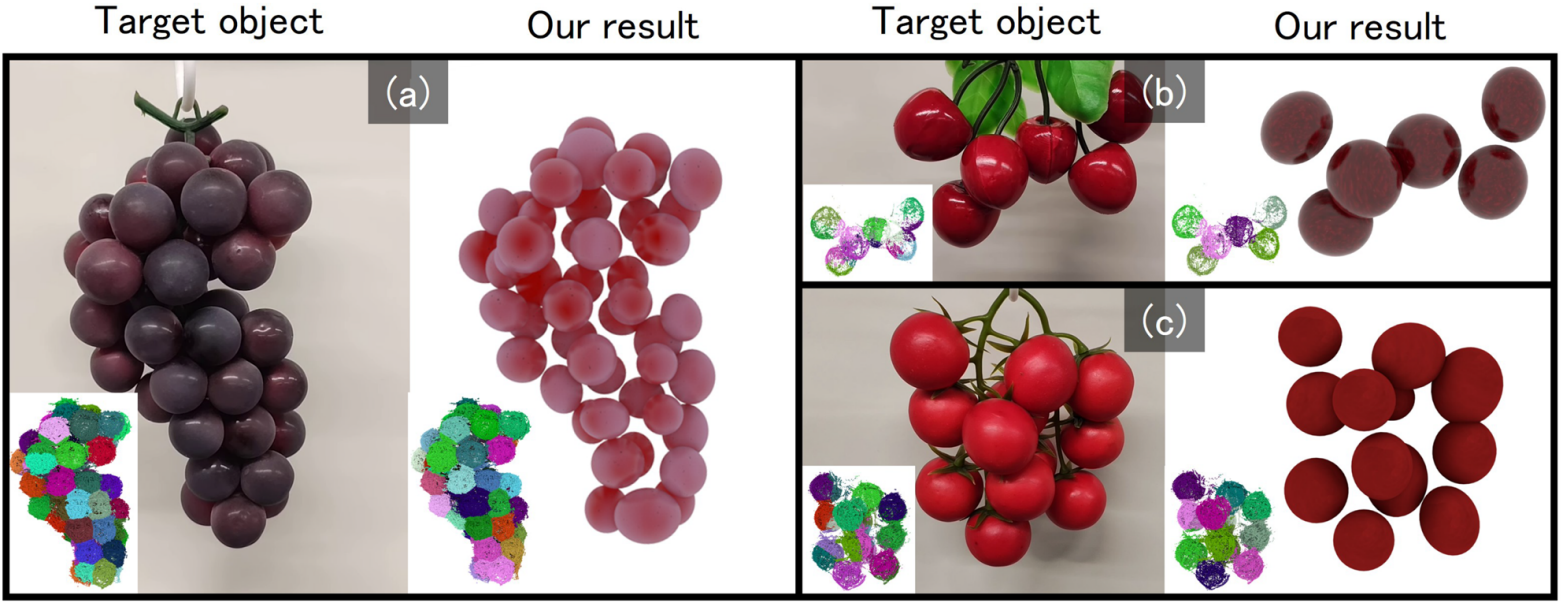
Our results on real food samples: (**a**) grape, (**b**) cherry, and (**c**) tomato. The inset image on the target object/our result shows initial/resultant clusters.

## Experimental results

Figures 5 and 6 show the results of aggregate modeling using the proposed method. Fig. 5 presents results obtained from point clouds generated from rendered images of aggregate 3D models using the 3DCG software Blender. Figure 6 shows results obtained from point clouds reconstructed from images of real food samples captured from 360 degrees around the object. Statistics of these experiments are summarized in Table 1. For point cloud generation from images, we used COLMAP^1^, a reconstruction system based on Structure from Motion. The image sets used as input to COLMAP were obtained as follows. For the rendered 3D models (Fig. 5), we placed a virtual camera at a fixed distance from the object and rendered 60 images by rotating the camera around the object in azimuth by 6 degrees increments over 360 degrees. When these 60 images did not yield a satisfactory point cloud reconstruction in COLMAP, we additionally rendered another 60 images with the virtual camera tilted by 35 degrees in elevation, resulting in a total of 120 images. For the real objects (Fig. 6), we suspended each sample from a small motorized rotating device (similar to a mirror ball motor) and captured a video using a smartphone while the object was continuously rotated. We then extracted approximately 90 frames from the recorded video for reconstruction. In our experiments, the images were captured under white fluorescent lighting; however, as long as the object is clearly visible, there are no strict constraints on illumination. For both the CG and real-image experiments, the image resolution was $1920 \times 1080$. After generating the point clouds, we applied color-based filtering and spatial cropping to extract only the point cloud corresponding to the target aggregate.

**Fig. 7:**
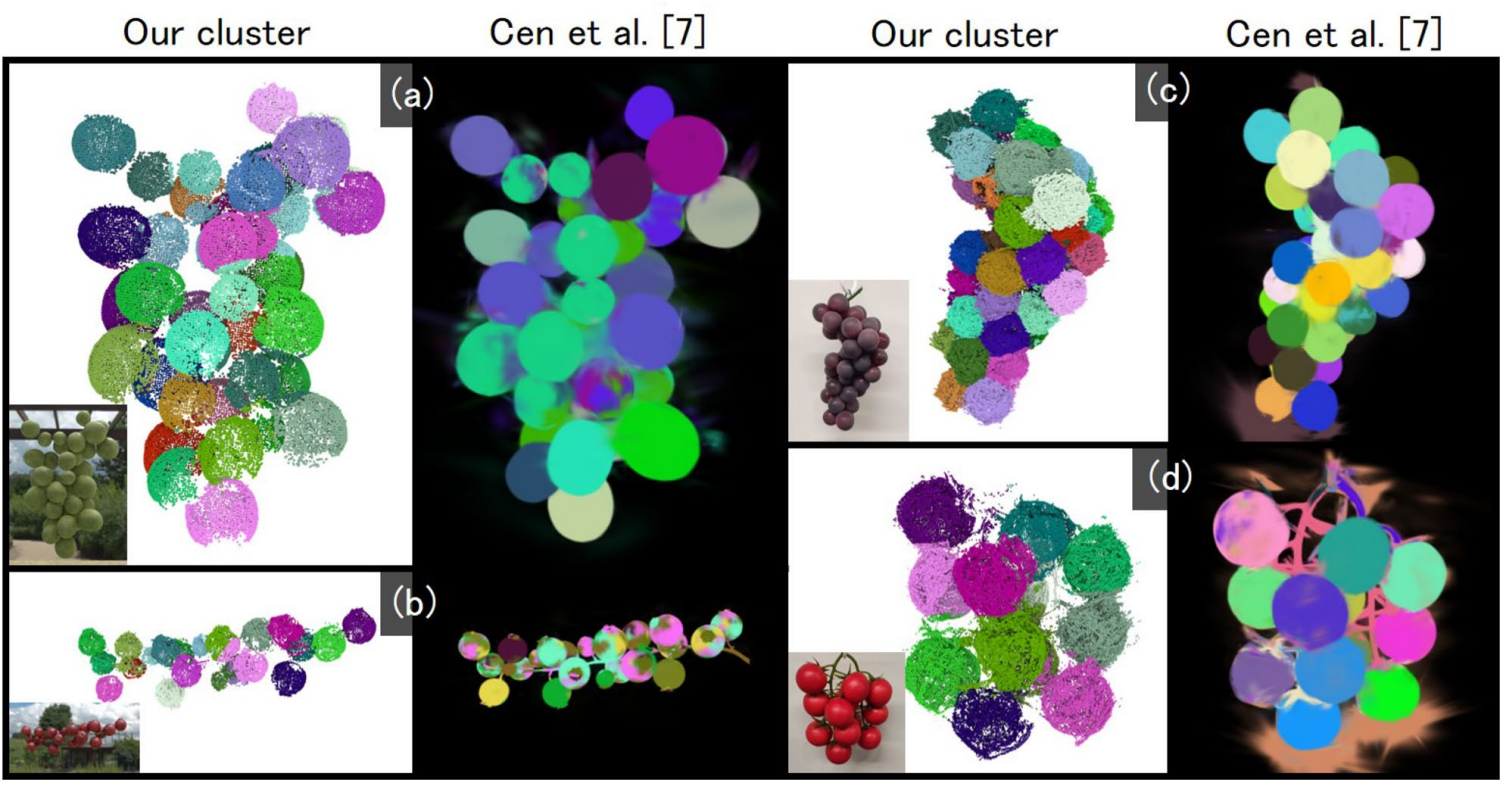
Comparisons with Segment Any 3D Gaussians. The inset image on our cluster shows the target object.

**Fig. 8:**
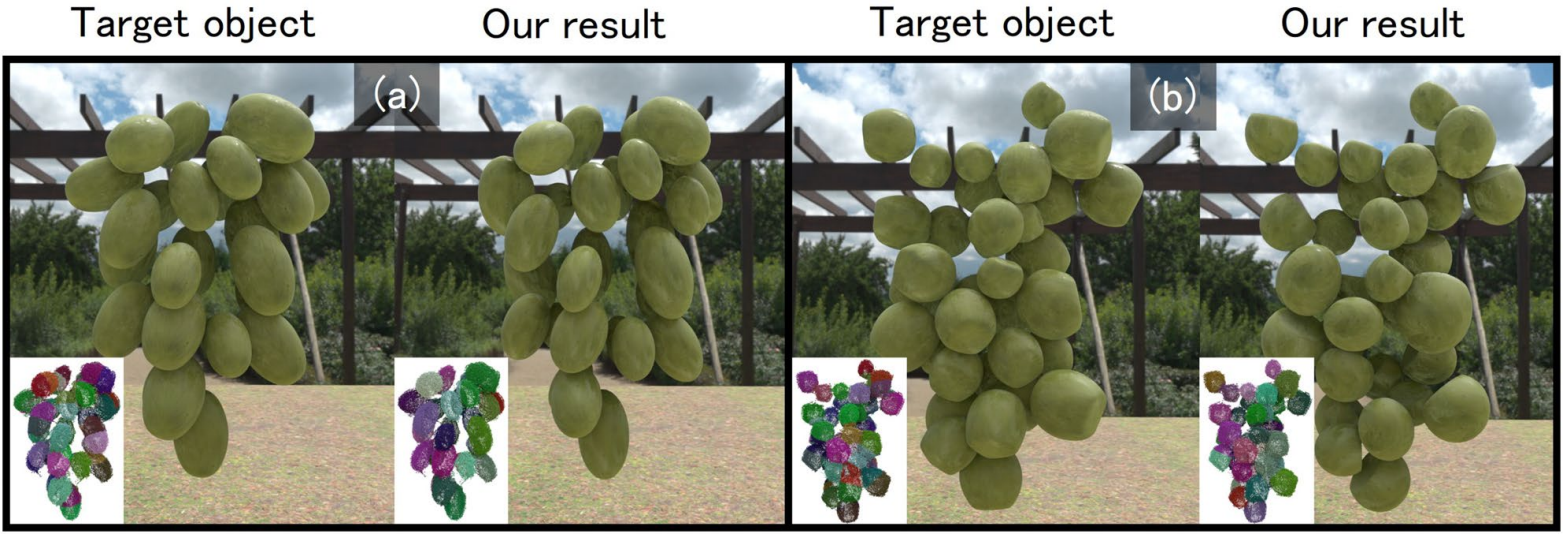
Our results on ellipsoidal and slightly deformed component shapes. The inset image on the target object/our result show initial/resultant clusters.

In the initial clusters (shown in the bottom-left corner of each “Target object” image), a single component is often split across multiple clusters. In contrast, after applying the proposed method, the clusters (shown in the bottom-left corner of each “Our result” image) are generally aligned with individual components. This is also supported by the small difference between the actual number of components $N_e$ and the number of clusters computed by our method $N_m$, as summarized in Table 1. Furthermore, by comparing the “Target object” and “Our result”, it is evident that the size and placement of most components are accurately estimated. The Supplementary video contains animation examples showing the point clouds and mesh models for each result, rendered from 360-degree viewpoints.

**Table 1:** Summary of the number of vertices in the input point clouds ($N_v$), initial number of clusters ($N_i$), actual number of components ($N_e$), number of clusters computed by our method ($N_m$), and computation time of our method ($t_o$, in seconds), for each result.

Experiment	$N_v$	$N_i$	$N_e$	$N_m$	$t_o$
Figure 5 (a)	55476	60	35	35	123.780
Figure 5 (b)	42913	60	24	24	136.966
Figure 5 (c)	44402	60	37	35	125.581
Figure 5 (d)	45198	100	69	72	183.46
Figure 5 (e)	21283	40	22	21	65.158
Figure 5 (f)	23293	20	8	8	164.165
Figure 6 (a)	117503	60	49	49	112.349
Figure 6 (b)	26123	15	6	6	84.72
Figure 6 (c)	123416	20	12	12	180.389
Figure 8 (a)	85075	60	27	27	1233.981
Figure 8 (b)	98846	60	37	35	1059.595

**Table 2:** Center-to-center mean distance ($M_d$) and standard deviation ($M_s$) between the input components and the corresponding components obtained by the proposed method for each result.

Experiment	$M_d$	$M_s$
Figure 5 (a)	0.020062	0.007476
Figure 5 (b)	0.030415	0.009254
Figure 5 (c)	0.027770	0.010666
Figure 5 (d)	0.022777	0.013357
Figure 5 (e)	0.020843	0.005546
Figure 5 (f)	0.034067	0.012271
Figure 8 (a)	0.021039	0.012019
Figure 8 (b)	0.017230	0.010914

Next, to quantitatively evaluate the quality of the results, we checked the accuracy of the positions of each aggregate component explored by the proposed method using CloudCompare^20^, similar to the conventional method^9^. Table 2 shows the results of calculating the mean and standard deviation of the center-to-center distances of corresponding components in the target 3D model and our result model. Each value is normalized based on the length of the diagonal of the bounding box covering the entire input model. In Table 2, the average center-to-center distance between corresponding components is around 0.02, and since the value of the conventional method^9^ is about 0.02-0.04, we achieved high accuracy in terms of position. The experiments were conducted on a PC equipped with an Intel Core i9-10850K processor and 64GB of memory. Excluding point cloud generation by COLMAP, the computation time for the proposed method was approximately 150 seconds. In contrast, the computation time excluding COLMAP in the conventional method^9^ has been reported to be at least 1,000 seconds. Although the computational environments used in the two studies are not identical and thus the times are not directly comparable, our method is more than six times faster. Moreover, since Woo et al.’s experiments were conducted using a GPU, it is reasonable to expect that our method, which has not yet been implemented on a GPU, could be even faster under similar conditions.

Next, Fig. 7 presents a comparison with the state-of-the-art method for 3D segmentation via Gaussian Splatting, namely SAGA^7^. SAGA enables interactive segmentation of scenes constructed using 3D Gaussian Splatting (3D-GS). The results obtained by SAGA are shown in the right column of Fig. 7, using the same set of input images as in our results. In all examples, SAGA produces several components that are fragmented into multiple small clusters, whereas our method does not exhibit such issues. SAGA relies on the Segment Anything Model (SAM)^21^, a 2D segmentation model used for training. However, SAM struggles to generate accurate masks for aggregates composed of many small, densely packed objects, such as those targeted in our study. As a result, SAGA, which uses these masks as supervision, fails to infer effective features for distinguishing individual components in 3D space. In contrast, our method achieves effective segmentation of aggregates through geometry-aware clustering.

In terms of comparison methods, the approach of Woo et al.^9^ is also based on a spherical assumption, as their framework approximates each grape berry as a sphere. SAGA^7^, on the other hand, is designed to handle more general 3D shapes. While fine-tuning SAGA on a dataset tailored to grape-like aggregates could potentially improve its segmentation accuracy in our setting, the training code for the model is not publicly available, which makes such experiments difficult to carry out. Furthermore, fine-tuning a large model would require a substantial amount of training data and compute. From the standpoint of computational and manual cost, we therefore consider our geometry-aware clustering approach to be advantageous for practical modeling of aggregates from point clouds.

Finally, We additionally evaluated the proposed method on aggregates composed of ellipsoidal and slightly deformed spherical components (Fig. 8). To support these non-spherical shapes, we made minimal but concrete extensions to the original pipeline, which had been designed under a spherical assumption. First, in the RANSAC-based fitting step, we generalized the inlier criterion. For each hypothesized component, represented as a mesh, we compute for every mesh vertex the distance to the nearest point in the corresponding cluster, and then calculate the proportion of vertices whose distance is below a predefined threshold. Second, in the hypothesis generation step, we extended the set of candidate components to include rotated variants. In addition to the unrotated hypothesis, we generate and evaluate patterns in which the component is rotated by $\pm 10$ degrees around each coordinate axis, in the same manner as in the post-fitting step. Third, we reformulated the cluster merging condition using inside–outside information. For a pair of hypothesized components, we compute the fraction of vertices of one component that lie inside the other (the number of vertices inside divided by the total number of vertices of that component). If this fraction exceeds a threshold *n*%, the two components are regarded as overlapping and are merged. In our experiments, a threshold of 2% yielded the best results for ellipsoidal components, while 10% worked best for the slightly deformed shapes. As shown in Fig. 8, Tables 1 and 2, the proposed method achieves good segmentation and localization performance even for these ellipsoidal and slightly deformed components under the extended settings. These results indicate that our framework can be naturally extended beyond purely spherical components and has the potential to handle more general component shapes.

## Discussion

In our method, the number of clusters *k* for k-means clustering and the initial radius of the component model must be specified by the user in advance. To investigate the range of these parameters within which the proposed method operates stably, we conducted a sensitivity analysis using the example in Fig. 5. For the cluster number, we considered three settings: $k=N_e$, $k=2N_e$, and $k=3N_e$, where $N_e$ is the actual number of components. When the components were relatively large (Fig. 5(a), (b), (c), and (f)), all three settings produced final cluster counts within approximately $\pm 2$ of $N_e$. However, for examples with smaller components (Fig. 5(d) and (e)), the settings $k=2N_e$ and $k=3N_e$ led to over-segmentation. These results suggest that choosing *k* to be about 1.5–2 times the actual number of components yields a stable final number of clusters. For the initial radius, we scaled the radius of the smallest visible component by factors of 0.7, 0.8, 0.9, 1.1, 1.2, and 1.3. Radii smaller than the smallest component (0.7–0.9) reduced the effectiveness of the merging step and consistently resulted in over-segmentation for all examples in Fig. 5. In contrast, larger radii (1.1–1.3) had little influence on the clustering quality and still produced appropriate segmentation. In terms of localization accuracy, the over-segmented cases showed degraded accuracy, whereas the other settings achieved accuracy comparable to that reported in Table 2. Automatic estimation of the cluster number *k* and the initial radius remains an important topic for future work. In particular, we expect that learning-based approaches, such as the deep learning framework of Woo et al.^9^, could be incorporated into our pipeline to predict appropriate parameter values from the input data.

Clustering could also, in principle, be performed using density-based methods; however, in our preliminary experiments with DBSCAN, it did not produce clusters as reliably as k-means, and therefore we did not adopt it in our framework. DBSCAN is highly sensitive to variations in point density, noise, and missing data due to occlusions. Point clouds reconstructed by SfM software such as COLMAP often exhibit inhomogeneous point density, with regions of both high and low density depending on the imaging conditions, and they may also contain noise. As a result, tuning the parameters (the neighborhood radius and the minimum number of samples) is difficult, and the resulting cluster shapes were less stable compared to those obtained with k-means. For example, when the neighborhood radius was set too large, almost the entire point cloud was labeled as a single cluster, whereas when the neighborhood radius was set too small, a mixture of a few large clusters and many small fragmented clusters appeared. Based on these observations, we concluded that k-means clustering is more suitable for our setting.

Although the proposed method significantly reduces search time compared to existing approaches, point cloud generation using COLMAP remains a bottleneck. In our experiments, 60 to 120 images were used to generate sufficiently dense point clouds for accurate component fitting, and the COLMAP reconstruction process alone took approximately 30 to 60 minutes. The conventional method proposed by Woo et al.^9^ also relies on COLMAP for point cloud reconstruction. In contrast, recent approaches using deep learning or Gaussian Splatting have the potential to reduce computational cost. However, in our preliminary experiments, applying the proposed method to point clouds generated by Gaussian Splatting did not yield satisfactory results. Therefore, it remains necessary to develop a faster point cloud reconstruction method that can maintain accuracy comparable to COLMAP.

**Fig. 9:**
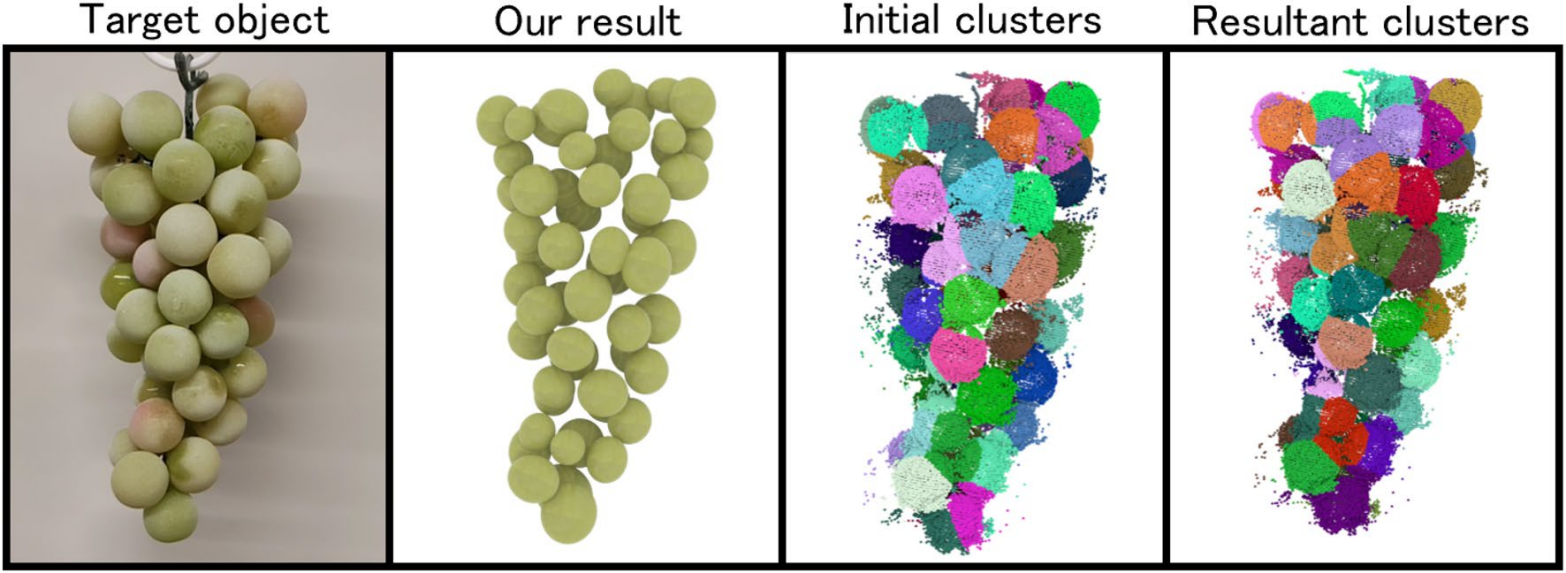
Failure case of our method.

In Fig. 5c, d and f, the size and placement of components are not accurately reconstructed in cases where the components are densely packed or extremely small. This is likely due to the fact that, in such densely packed regions, the point clouds corresponding to individual components cannot be fully captured from the input images. This issue is particularly evident in the example shown in Fig. 9, where several clusters are not correctly identified. In this case, while the actual number of components is 49, the number of clusters obtained by the proposed method is 56, which resulted in over-segmentation compared to other examples. These observations suggest that future work should consider incorporating approaches capable of inferring occluded or unobserved parts of the components from images.

## Conclusions

In this paper, we proposed a method for modeling an aggregate from images, with the goal of arranging components to match input images and creating independent mesh models for each component. We first extract a point cloud of an aggregate from the images with COLMAP. By applying k-means clustering to the point clouds and repeatedly performing fitting and merging on the obtained clusters, we created mesh models with separated components of the aggregate. The proposed method was able to maintain position accuracy equivalent to conventional methods while achieving faster processing times.

One important direction for future work is to improve the accuracy of the fitting process. The current method assumes that the point cloud accurately captures the geometry of the components; however, in practice, point clouds may suffer from missing shape data due to occlusions in the input images, as well as varying point density and noise caused by measurement conditions. Therefore, it is necessary to develop more robust fitting algorithms and incorporate methods that can predict the shapes of occluded or missing regions. Another challenge lies in handling a wider variety of component shapes. The current method targets only aggregates composed of spherical components, but real-world aggregates often contain elements with diverse shapes, such as ellipsoids or irregular geometries. By improving the clustering and fitting procedures, we aim to extend the method to support more general types of aggregates.

## Supplementary Information

Supplementary Information.

## Data Availability

The data generated during the current study available from the corresponding author on reasonable request.
